# Reference Values of Cardiopulmonary Exercise Test Parameters in the Contemporary Paediatric Population

**DOI:** 10.1186/s40798-023-00622-3

**Published:** 2023-08-01

**Authors:** Pascal Amedro, Stefan Matecki, Taissa Pereira dos Santos, Sophie Guillaumont, Jonathan Rhodes, Suellen Moli Yin, Alfred Hager, Julia Hock, Gregoire De La Villeon, Johan Moreau, Anne Requirand, Luc Souilla, Marie Vincenti, Marie-Christine Picot, Helena Huguet, Thibault Mura, Arthur Gavotto

**Affiliations:** 1 Department of Paediatric and Congenital Cardiology, M3C National Reference Centre, Bordeaux University Hospital, 1 Avenue Magellan, 33604 Pessac, France; 2 IHU Liryc, Electrophysiology and Heart Modelling Institute, INSERM 1045, Bordeaux University Foundation, Avenue du Haut Lévêque, 33600 Pessac, France; 3 PhyMedExp, CNRS, INSERM, University of Montpellier, 371 Avenue du Doyen Giraud, 34295 Montpellier, France; 4 Paediatric Functional Exploration Laboratory, Physiology Department, Montpellier University Hospital, 371 Avenue du Doyen Giraud, 34295 Montpellier, France; 5 Department of Biostatistics, Clinical Epidemiology, Public Health, and Innovation in Methodology, Nimes University Hospital, University of Montpellier, Place du Professeur Debré, 30029 Nimes, France; 6 Department of Paediatric and Congenital Cardiology, M3C Regional Reference CHD Centre, Montpellier University Hospital, 371 Avenue du Doyen Giraud, 34295 Montpellier, France; 7 Paediatric Cardiology and Rehabilitation Unit, St-Pierre Institute, 371 Avenue de L’Évêché de Maguelone, 34250 Palavas-Les-Flots, France; 8 Department of Cardiology, Boston Children’s Hospital, 300 Longwood Avenue, Boston, MA 02115 USA; 9 Clinic for Paediatric Cardiology and Congenital Heart Diseases, German Heart Centre, Lazarettstrasse 36, 80636 Munich, Germany; 10 Clinical Research and Epidemiology Unit, Montpellier University Hospital, INSERM-CIC 1411, Clinical Investigation Centre, University of Montpellier, Montpellier, France; 11 INSERM, U1061, Neuropsychiatry: Epidemiological and Clinical Research, University of Montpellier, 39 Av. Charles Flahault, 34090 Montpellier, France; 12 Paediatric Intensive Care Unit, Arnaud de Villeneuve Hospital, Montpellier University Hospital, 371 Avenue du Doyen Giraud, 34295 Montpellier, France; 13 Department of Paediatric and Adult Congenital Cardiology, M3C National CHD Reference Centre, Bordeaux University Hospital, Haut-Leveque Hospital, Avenue de Magellan, 33604 Pessac Cedex, France

**Keywords:** Physical fitness, Aerobic exercise, Maximal breathing capacity, Children, Z-score

## Abstract

**Background:**

The evaluation of health status by cardiopulmonary exercise test (CPET) has shown increasing interest in the paediatric population. Our group recently established reference Z-score values for paediatric cycle ergometer VO_2max_, applicable to normal and extreme weights, from a cohort of 1141 healthy children. There are currently no validated reference values for the other CPET parameters in the paediatric population. This study aimed to establish, from the same cohort, reference Z-score values for the main paediatric cycle ergometer CPET parameters, apart from VO_2max_.

**Results:**

In this cross-sectional study, 909 healthy children aged 5–18 years old underwent a CPET. Linear, quadratic, and polynomial mathematical regression equations were applied to identify the best CPET parameters Z-scores, according to anthropometric parameters (sex, age, height, weight, and BMI). This study provided Z-scores for maximal CPET parameters (heart rate, respiratory exchange ratio, workload, and oxygen pulse), submaximal CPET parameters (ventilatory anaerobic threshold, VE/VCO_2_ slope, and oxygen uptake efficiency slope), and maximum ventilatory CPET parameters (tidal volume, respiratory rate, breathing reserve, and ventilatory equivalent for CO_2_ and O_2_).

**Conclusions:**

This study defined paediatric reference Z-score values for the main cycle ergometer CPET parameters, in addition to the existing reference values for VO_2max_, applicable to children of normal and extreme weights. Providing Z-scores for CPET parameters in the paediatric population should be useful in the follow-up of children with various chronic diseases. Thus, new paediatric research fields are opening up, such as prognostic studies and clinical trials using cardiopulmonary fitness outcomes.

*Trial registration* NCT04876209—Registered 6 May 2021—Retrospectively registered, https://clinicaltrials.gov/ct2/show/NCT04876209.

**Supplementary Information:**

The online version contains supplementary material available at 10.1186/s40798-023-00622-3.

## Key Points

- The dissemination of CPET in paediatrics requires valid paediatric reference values to define the upper and lower normal limits for the main parameters, in addition to the existing reference values for VO_2max_.
- The paediatric cycle ergometer CPET reference values were generated using the best mathematical model for the Z-score construction, according to the main anthropometric predictors (sex, age, height, weight, and BMI).
- Providing Z-scores for the main cycle ergometer CPET parameters in the paediatric population will be useful in the follow-up of children with chronic diseases.

**Supplementary Information:**

The online version contains supplementary material available at 10.1186/s40798-023-00622-3.

## Background

There has been increasing interest in evaluating the health status of children by cardiopulmonary exercise test (CPET) [1]. In many paediatric chronic diseases, impaired physical capacity assessed by CPET correlates with lower health-related quality of life scores and may indicate the early onset of physical deconditioning, such as in congenital heart disease (CHD) [2], cancer [3], asthma [4], or kidney disease [5]. Therefore, CPET stands as a key examination to evaluate cardiopulmonary fitness in healthy and chronically ill children [6], as well as to promote physical activity and cardiovascular rehabilitation from a young age [7].

The VO_2max_ is the main CPET parameter to evaluate the level of physical capacity, and an independent predictor of cardiovascular risk [8]. The VO_2max_ reference values defined by Cooper et al. in 1984, are based on linear equations from a small cohort of 109 healthy children with normal weight and are currently less adapted to the contemporary paediatric population [9, 10]. Our group recently established reference Z-score values for paediatric cycle ergometer VO_2max_, applicable to normal and extreme weights, from a cohort of 1141 healthy children, including 909 children in the development cohort and 232 children in the validation cohort [11]. For both sexes, the Z-score equations were modelled with a logarithmic function of VO_2max_, height, and BMI.

While reference values have been established for VO_2max_, there are currently no validated reference values for the other main CPET parameters. As recently stated by Takken et al., “there is still a lot of progress to be made” in validating reference equations in paediatric CPET [12]. Yet, the other CPET parameters are complementary to VO_2max_ to assess cardiac, muscular and respiratory functions. In particular, greater attention has been given to submaximal parameters in paediatric CPET: the ventilatory anaerobic threshold (VAT) reflects the level of muscular deconditioning and has been used to determine the intensity of physical activity in paediatric cardiovascular rehabilitation programs [7]; the ventilatory efficiency slope (e.g. VE/VCO2 slope) increases in paediatric heart failure, especially in the most complex heart diseases [13]; and the oxygen uptake efficiency slope (OUES) may be used as a surrogate of VO_2max_ in children unable to perform a maximal exercise test [14].

Therefore, the dissemination of CPET in children requires contemporary paediatric reference values, based on valid mathematical models to define the upper and lower normal limits for each CPET parameter.

In this study, we aimed to establish paediatric reference Z-score values for the main cycle ergometer CPET parameters, apart from VO_2max_, from a large cohort of healthy children representative of the contemporary paediatric population, including extreme weights.

## Methods

### Study Design and Population

To elaborate paediatric CPET parameters Z-scores, we used a French CPET database, initially generated by pooling all subjects aged less than 18 years enrolled in previous prospective controlled studies carried out in two paediatric CPET laboratories (centre 1: M3C Regional Paediatric and Congenital Cardiology Centre, Montpellier University Hospital, France; centre 2: Paediatric Cardiology and Rehabilitation Centre, Saint-Pierre Institute, Palavas-Les-Flots, France) [2, 7, 13–18]. We identified all subjects who underwent a complete CPET with a high-quality score (≥ 10 points) according to the ATS/ACCP statement [19], over a period of 10 years (from November 2010 to March 2020).

Children referred for CPET with the following clinical criteria were selected: non-severe functional symptoms at rest (murmur, palpitation) or during exercise (chest pain or dyspnoea), and completely normal cardiological check-up, including physical examination, electrocardiogram, and echocardiography. Children with overweight or obesity who were referred to the CPET laboratory for physical fitness check-up were also eligible for the study. However, children with any other chronic disease, medical condition (cardiac, neurological, respiratory, muscular, or renal), or medical treatment and those requiring any further specialized medical consultation were not eligible.

Anthropometric parameters were collected (sex, age, weight, and height) and body mass index (BMI) percentiles were used to define patient groups: underweight (BMI < 5th percentile), normal weight (BMI between 5 and 84th), and overweight or obesity (BMI ≥ 85th) [20].

### CPET Procedures

CPET procedures for children enrolled in the study were harmonized and similar in both laboratories [2, 13, 14, 16]. We used a single CPET paediatric cycle ergometer protocol adapted to children to obtain a homogeneous incremental overall duration between 8 to 12 min including (a) 1-min baseline; (b) 3-min warm-up (10 or 20 watts), (c) fixed increments of 10, 15, or 20 watts each minute, (d) pedalling rate of 60–80 revolutions per minute, (e) 3-min active recovery (20 watts); f) 2-min rest [21]. The CPET was considered maximal when the child was unable to maintain a pedalling rate above 60 despite verbal encouragement. When the VO_2max_ did not reach a plateau, the peak VO2 value was collected, as usual in paediatrics [22, 23].

Spirometry (flow volume curve) was performed at rest to measure the forced expiratory volume in 1 s (FEV1), the forced vital capacity (FVC), and the FEV1/FVC ratio (FEV1/FVC%) [24].

From 2010 to 2015, the same technical devices were used in both CPET laboratories: paediatric face masks (Hans Rudolph, Shawnee, KS, USA), calibrated gas analyser (Oxycon Pro, Jaeger, Erich Jaeger GmbH, Hoechberg, Germany), breath-to-breath measurement software (Windows 98, Jaeger), 12-lead ECG equipment (CardioSoft, GE Healthcare, Little Chalfont, UK), pulse oximeter (Nellcor, Medtronic, Fridley, MN, USA), and manual sphygmomanometer with adapted paediatric cuffs. From 2015, the centre 1 used the following technical devices: calibrated gas analyser (Quark CPET, Cosmed Srl, Pavonna di Albano, Italy), breath-by-breath measurement software (Windows 7–10, Omnia, Cosmed), 12-lead ECG equipment (Norav, Germany), and pulse oximeter (Nonin Medical Inc, Plymouth, MN 55441 USA).

### CPET Parameters

Apart from VO_2max_, the CPET parameters were grouped into 3 categories: maximal parameters, submaximal parameters, and ventilatory parameters.

The maximal CPET parameters included: (1) the maximal heart rate; (2) the maximal respiratory exchange ratio (RER_max_), which corresponds to the ratio of CO_2_ elimination (VCO_2_) and oxygen uptake (VO_2_); (3) the maximum workload in Watts (maintained for at least 30 s by the patient); (4) and the maximum oxygen pulse (O_2_ pulse_max_), e.g. a surrogate of stroke volume at peak exercise, which corresponds to the ratio between VO_2max_ and maximal heart rate [19].

The submaximal CPET parameters included: (1) the VAT, e.g. the point at which minute ventilation increases disproportionally relative to VO_2_, which reflects muscular response to exercise [2, 25], and manually calculated by a single investigator using V-slope method [26] and expressed as a percentage of the predicted VO_2max_ (%-predicted VAT) [11]; (2) the VE/VCO_2_ slope, e.g. an indicator of ventilation-perfusion ratio during exercise [13, 27], calculated from breath-by-breath data using linear regression of minute ventilation (VE) and CO_2_ production (VCO_2_) obtained during incremental exercise (VE = [VE/VCO_2_ slope] × VCO_2_ + b) [13], and measured from the beginning of incremental exercise (after the warm-up period) to maximum exercise (or respiratory compensation point when present) [2, 13]; (3) and the OUES, e.g. a submaximal surrogate parameter of VO_2max_, calculated from breath-by-breath data using linear regression of VO_2_ on logarithmically converted VE (VO_2_ = OUES × log_10_ VE + b), measured from the beginning of the exercise test to its maximal point, and expressed as a weight-normalized value (OUES_Kg_) [14].

The ventilatory parameters included: (1) the maximum tidal volume (VT_max_), which corresponds to the patient's inspiratory volume for each breath at maximum effort averaged over 30 s [19]; (2) the maximum respiratory rate (RR_max_), which corresponds to the respiratory rate averaged over 30 s at maximum effort [19]; (3) the breathing reserve (BR), calculated using the formula BR (%) = [MVV − VE_max_] ÷ MVV*100 (with MVV, maximal voluntary ventilation = 35*FEV1) [19]; (4) the maximum ventilatory equivalent for CO_2_ (VEqCO_2max_) which corresponds to the ratio between VE and VCO_2_ averaged over 30 s at maximum effort [28]; and (5) the maximum ventilatory equivalent for O_2_ (VEqO_2max_) which corresponds to the ratio between the VE and the VO_2_ averaged over 30 s at maximum effort [19].

### Statistics

The study population was described using means and standard deviations (SD) for quantitative variables and with frequencies for qualitative variables. Quantitative variables were compared using Student's t-test when the distribution was Gaussian and using the Mann–Whitney test otherwise. For qualitative variables, groups were compared using the chi-squared test or Fisher’s exact test.

To identify the best model for the Z-score construction, the values of each CPET parameter were modelized according to the main anthropometric predictors: sex, age, height, weight, and BMI. Three different mathematical regression models were successively applied: (1) a linear model, (2) a quadratic model, and (3) a polynomial model of degree 2 [29]. Models with several potential predictors were also evaluated. The models maximizing Pearson's coefficient of determination (*R*^2^, e.g. the proportion of variance of each CPET parameter explained by the model) were identified. To generate a model respecting the condition of homoscedasticity required for the Z-score construction, we also evaluated all linear, quadratic, and polynomial models with log–log relationships (e.g.*,* modelling the natural logarithm of the CPET parameters with the natural logarithm of the anthropometric predictor(s)). Homoscedasticity was assessed by comparing the standard deviation of the estimated model residuals between quintiles of the predicted value. Finally, all the models selected met this condition. The data were first analysed for all sexes. A sex effect and an interaction between sex and anthropometric predictor(s) were then tested in the selected model. When one of these effects was significant, separate models were performed for boys and girls. For each gender, we finally selected the model with the highest *R*^2^ and the lowest heteroscedasticity in the residuals [30]. When the complexity of the model (quadratic, polynomial or multivariate) only allowed a gain of less than 0.01 point of *R*^2^, the simplest model was chosen. When no anthropometric predictor explained more than 5% of the variability of the parameter of interest (e.g. *R*^2^ < 0.05 for all models), a simple Z-score, obtained by subtracting the mean and dividing by the standard deviation, was constructed after checking graphically that the distribution was close to the Gaussian distribution. The final Z-score distribution was described using median, 5th, and 95th percentiles. Under the condition of standard normal distribution, the expected values of the Z-score are 0, -1.64, and 1.64, respectively.

Statistical analyses were performed at the conventional two-tailed α level of 0.05 using SAS version 9.4 (SAS Institute, Cary, NC).

## Results

### Population

During the 10-year study period, a total of 909 children aged 5–18 (mean age at 11.4 ± 2.7 years, 477 boys and 432 girls), following the eligibility criteria, were selected from the CPET database, and included in the study. The numbers of patients per age group were *N* = 142 (*i.e.* 60 girls and 82 boys) [5–8 years], *N* = 333 (*i.e.* 156 girls and 177 boys) [9–11 years], *N* = 317 (*i.e.* 153 girls and 164 boys) [12–14 years], and *N* = 117 (*i.e.* 63 girls and 54 boys) [15–18 years]. After stratification on BMI, we identified 38 underweight children, 639 children with normal weight, and 232 children with overweight or obesity (of which 81 children with overweight ≥ 85th percentile and 151 children with obesity ≥ 95th percentile). No significant differences between boys and girls were observed in terms of age, height, weight, and BMI. The main anthropometric data of the population are summarized in Table 1.

**Table 1:** Main anthropometric and CPET data

Parameters	Variables	All	Girls	Boys	*P*-value*
	*N*	909	432	477	
Anthropometry	Age (years)	11.4 ± 2.7	11.5 ± 2.7	11.2 ± 2.6	0.06
	Height (cm)	151.2 ± 15.5	151.2 ± 14.2	151.3 ± 16.6	0.90
	Weight (Kg)	47.9 ± 21.1	48.3 ± 19.8	47.5 ± 22.3	0.57
	BMI (Kg/m^2^)	20.3 ± 6.2	20.6 ± 6.2	20.0 ± 6.2	0.13
	BMI (percentile)	56.6 ± 30.8	57.9 ± 30.3	55.4 ± 31.2	0.22
	BMI ≥ 85th percentile (*n*)	232	116	116	0.42
	Sex ratio (male/female)	1.1	–	–	
Spirometry	FEV1 (Z-score)	0.02 ± 1.16	− 0.10 ± 1.06	0.12 ± 1.22	**< 0.01**
	FVC (Z-score)	0.08 ± 1.27	0.01 ± 1.16	0.14 ± 1.37	0.14
	FEV1/FVC (Z-score)	− 0.06 ± 1.15	− 0.18 ± 1.10	0.04 ± 1.18	**0.01**
Maximal parameters	VO_2max_ (mL/min)	1794 ± 556	1639 ± 427	1934 ± 620	**< 0.01**
	VO_2max_ (mL/Kg/min)	40.0 ± 9.4	36.1 ± 7.9	43.5 ± 9.3	**< 0.01**
	HR_max_ (bpm)	188.1 ± 8.9	188.1 ± 8.9	188.2 ± 8.8	0.84
	RER_max_	1.15 ± 0.11	1.16 ± 0.12	1.14 ± 0.10	**< 0.01**
	Maximum workload (Watts)	141 ± 50	129 ± 38	153 ± 56	**< 0.01**
	O_2_ pulse_max_ (mL)	9.6 ± 3.0	8.7 ± 2.4	10.3 ± 3.3	**< 0.01**
Submaximal parameters	VAT (mL/min)	1254 ± 386	1148 ± 315	1350 ± 419	**< 0.01**
	VAT (mL/Kg/min)	28.1 ± 7.2	25.4 ± 6.3	30.5 ± 7.1	**< 0.01**
	%-Predicted VAT (%)	70.4 ± 9.0	70.4 ± 9.5	70.4 ± 8.5	0.97
	VE/VCO_2_ slope	30.4 ± 4.3	30.6 ± 4.5	30.1 ± 4.2	0.1
	OUES	1943 ± 636	1776 ± 526	2086 ± 685	**< 0.01**
	OUES_kg_	42.4 ± 11.4	38.0 ± 9.7	46.1 ± 11.4	**< 0.01**
Ventilatory parameters	VT_max_ (L)	1.34 ± 0.48	1.28 ± 0.42	1.40 ± 0.53	**< 0.01**
	VT_max_ (mL/Kg)	29.4 ± 7.1	27.8 ± 6.5	30.9 ± 7.3	**< 0.01**
	RR_max_ (/min)	50.2 ± 10.0	49.2 ± 9.8	51.1 ± 10.2	**< 0.01**
	Breathing reserve (%)	26.4 ± 14.7	28.7 ± 15.5	24.3 ± 13.7	**< 0.01**
	VEqCO_2max_	30.8 ± 3.7	31.0 ± 3.8	30.6 ± 3.6	0.12
	VEqO_2max_	35.5 ± 5.6	36.2 ± 5.8	35.0 ± 5.3	**< 0.01**

Values are mean ± standard deviation

Significant *P*-values are marked in bold

*BMI*—body mass index; *CPET*—cardiopulmonary exercise test; *FEV1*—forced expiratory volume in 1 s; *FVC*—forced vital capacity; *FEV1/FVC*—Tiffeneau index; *O*_*2*_* pulse*_*max*_—maximum oxygen pulse; *OUES*—oxygen uptake efficiency slope; *RER*_*max*_—maximum respiratory exchange ratio; *RR*_*max*_—maximum respiratory rate; *VAT*—ventilatory anaerobic threshold; %-*predicted VAT*—VAT expressed as a percentage of the predicted VO_2max_; *VEqCO*_*2max*_—maximum ventilatory equivalent for CO_2_; *VEqO*_*2max*_—maximum ventilatory equivalent for O_2_; *VT*_*max*_—maximum tidal volume

*Comparison between girls and boys

### CPET Parameters

Overall, this study fulfilled 11 of the 14 criteria from the ATS/ACCP statement on cardiopulmonary exercise testing (Additional file 1: Table S1).

Significant sex differences were observed in most CPET parameters, except for VE/VCO_2_ slope, VEqCO_2max_, maximum heart rate, and %-predicted VAT (Table 1). VAT, OUES, O_2_ pulse_max_, and VT_max_ were mainly influenced by height and weight, the maximum workload was influenced by height and age, and the VE/VCO_2_ slope was slightly influenced by age. Maximum heart rate, breathing reserve, and VEqCO_2max_ were not influenced by any anthropometric parameters. The CPET parameters with the highest coefficients of determination (*R*^2^) according to anthropometric data were reported in Additional file 1: Table S2.

### CPET Parameters Z-Scores

**Fig. 1:**
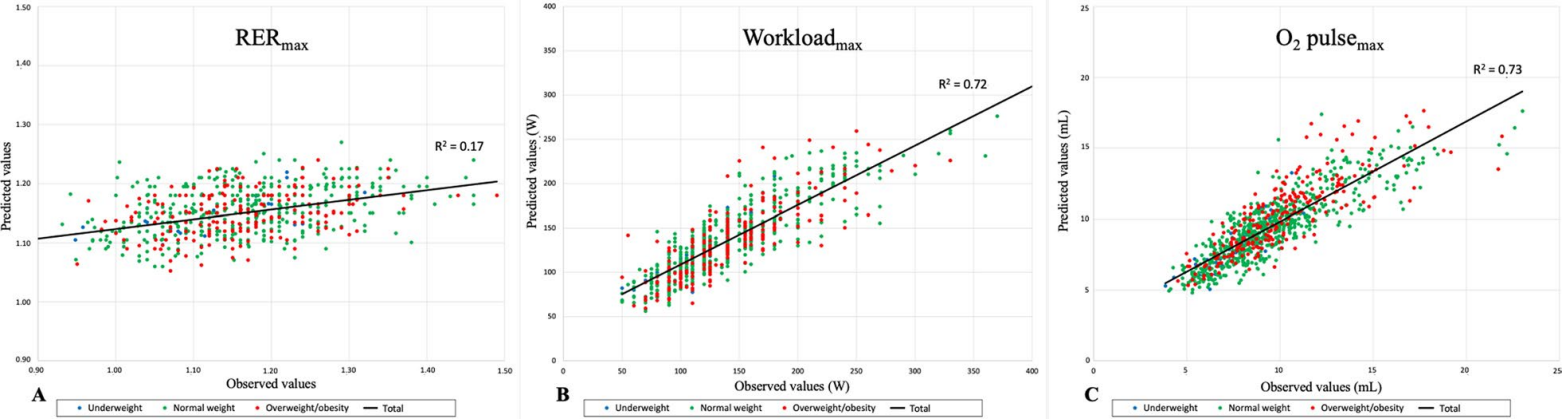
Correlation between observed and predicted values of CPET maximal parameters using the Z-score model. The “underweight” group was represented by blue points, the “normal weight” group by green points, and the “overweight/obesity” group by red points. The correlation between measured and predicted values using the Z-score model for RER_max_ (panel **A**), workload_max_ (panel **B**) and O_2_ pulse_max_ (panel **C**)

**Fig. 2:**
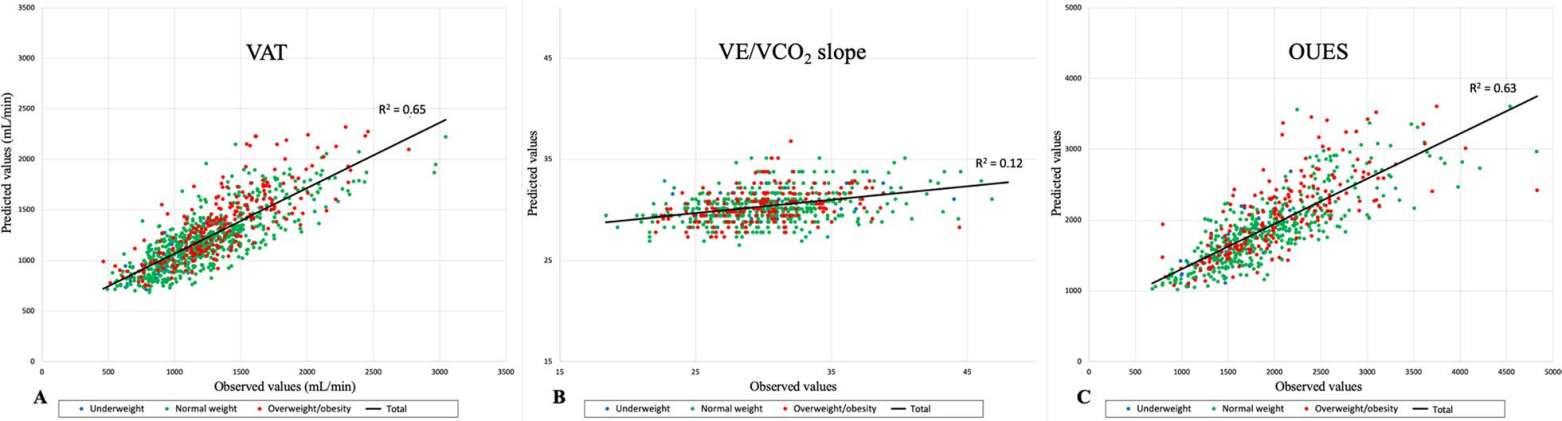
Correlation between observed and predicted values of CPET submaximal parameters using the Z-score model. The “underweight” group was represented by blue points, the “normal weight” group by green points, and the “overweight/obesity” group by red points. The correlation between measured and predicted values using the Z-score model for VAT (panel **A**), VE/VCO_2_ slope (panel **B**) and OUES (panel **C**)

**Fig. 3:**
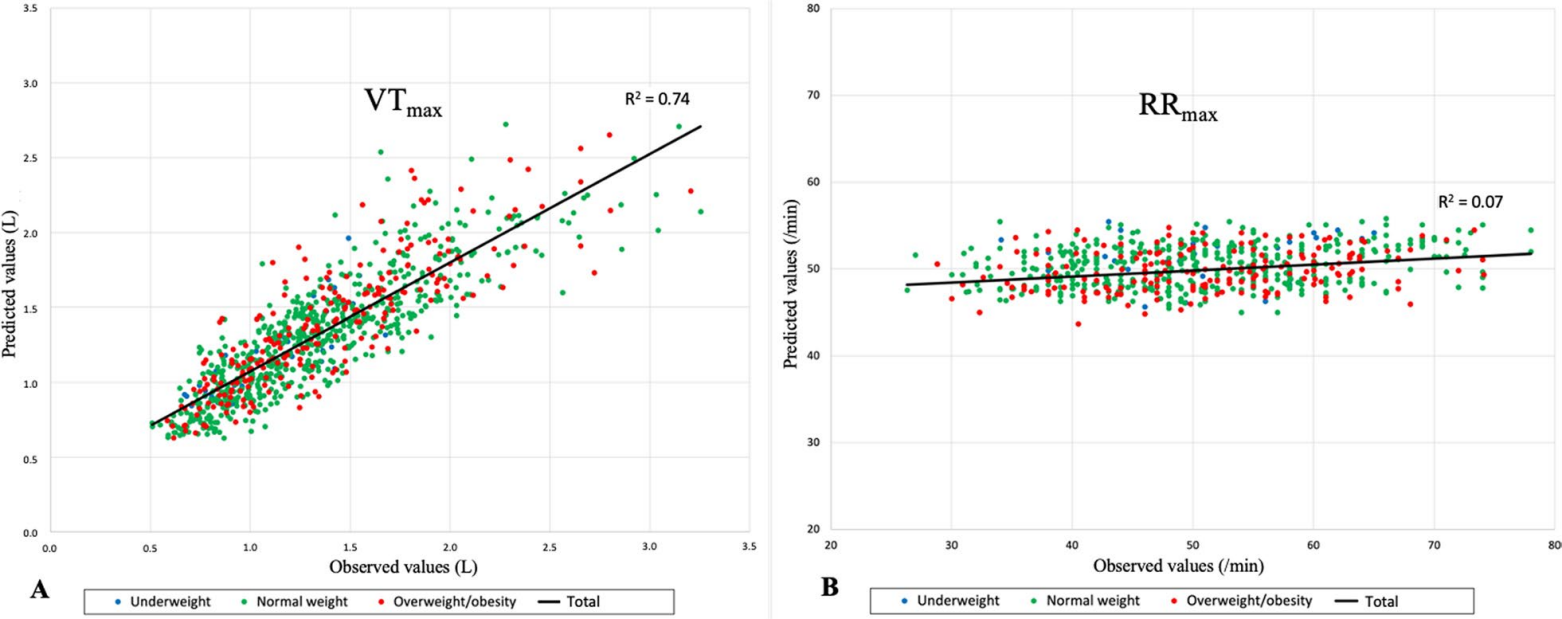
Correlation between observed and predicted values CPET ventilatory parameters using the Z-score model. The “underweight” group was represented by blue points, the “normal weight” group by green points, and the “overweight/obesity” group by red points. The correlation between measured and predicted values using the Z-score model for VT_max_ (panel **A**) and RR_max_ (panel **B**)

Table 2 summarizes the equations for each CPET parameter Z-score and reported the variability of each parameter logarithm (*R*^2^), including median, 5th, and 95th Z-score percentiles. Figures 1, 2, and 3 summarize the correlation between observed and predicted values, distinguishing the different BMI groups (underweight, normal weight, and overweight/obesity). The median CPET values with their ranges [5th percentile; 95th percentile] in the normal weight population were presented in Additional file 1: Table S3.

**Table 2:** Z-score equations of CPET parameters

CPET parameters	Sex	Z-score equations	*N*	*R*^2^	5th percentile	Median	95th percentile
HR_max_ (bpm)	All gender	${\text{Z - score}} = \frac{{\left( {{\text{HR}}_{{{\text{max}}}} \left( {{\text{bpm}}} \right) - 188.1} \right)}}{8.9}$	909	–	− 1.70	− 0.01	1.67
RER_max_	Girls	${\text{Z - score}} = \frac{{{\text{RER}} - 0.985 - 0.015 \times {\text{age}}_{{\left( {{\text{years}}} \right)}} }}{0.098}$	431	0.14	− 1.46	0.01	1.73
	Boys	${\text{Z - score}} = \frac{{\ln_{{{\text{RER}}}} + 2.01 - 0.475 \times \ln_{{{\text{height}}\left( {{\text{cm}}} \right)}} + 0.064 \times \ln_{{{\text{weight}}\left( {{\text{kg}}} \right)}} }}{0.0769}$	477	0.16	− 1.79	0.08	1.44
Workload_max_ (Watts)	Girls	${\text{Z - score}} = \frac{{\ln_{{{\text{load}}}} + 5.951 - 1.947 \times \ln_{{{\text{height}}\left( {{\text{cm}}} \right)}} - 0.39 \times \ln_{{{\text{age}}\left( {{\text{years}}} \right)}} }}{0.1932}$	390	0.60	− 1.61	− 0.02	1.58
	Boys	${\text{Z - score}} = \frac{{\ln_{{{\text{load}}}} + 7.662 - 2.309 \times \ln_{{{\text{height}}\left( {{\text{cm}}} \right)}} - 0.408 \times \ln_{{{\text{age}}\left( {{\text{years}}} \right)}} }}{0.1713}$	419	0.76	− 1.82	0.09	1.66
O_2_ pulse_max_ (mL)	Girls	${\text{Z - score}} = \frac{{\ln_{{02{\text{pulse}}}} + 6.266 - 1.049 \times \ln_{{{\text{weight}}\left( {{\text{kg}}} \right)}} + 0.091 \times \ln_{{{\text{weight}}\left( {{\text{kg}}} \right)}} \times \ln_{{{\text{weight}}\left( {{\text{kg}}} \right)}} - 1.147 \times \ln_{{{\text{height}}\left( {{\text{cm}}} \right)}} }}{0.1484}$	432	0.69	− 1.66	− 0.04	1.82
	Boys	${\text{Z - score}} = \frac{{\ln_{{02{\text{pulse}}}} + 8.04 - 0.15 \times \ln_{{{\text{weight}}\left( {{\text{kg}}} \right)}} - 1.946 \times \ln_{{{\text{height}}\left( {{\text{cm}}} \right)}} }}{0.1458}$	477	0.77	− 1.64	− 0.01	1.60
VAT (mL/min)	Girls	${\text{Z - score}} = \frac{{\ln_{{{\text{VAT}}\left( {\text{mL/min}} \right)}} - 1.603 - 0.7875 \times \ln_{{{\text{height}}\left( {{\text{cm}}} \right)}} - 0.3824 \times \ln_{{{\text{weight}}\left( {{\text{kg}}} \right)}} }}{0.1872}$	432	0.54	− 1.55	− 0.02	1.73
	Boys	${\text{Z - score}} = \frac{{\ln_{{{\text{VAT}}\left( {\text{mL/min}} \right)}} + 1.5616 - 1.5681 \times \ln_{{{\text{height}}\left( {{\text{cm}}} \right)}} - 0.228 \times \ln_{{{\text{weight}}\left( {{\text{kg}}} \right)}} }}{0.1714}$	477	0.68	− 1.83	− 0.08	1.65
VE/VCO_2_ slope	Girls	${\text{Z - score}} = \frac{{\ln_{{\frac{{{\text{VE}}}}{{{\text{VCO}}_{2} }}{\text{slope}}}} - 3.80 + 0.158 \times \ln_{{{\text{age}}\left( {{\text{years}}} \right)}} }}{0.1411}$	423	0.06	− 1.61	0.03	1.74
	Boys	${\text{Z - score}} = \frac{{\ln_{{\frac{{{\text{VE}}}}{{{\text{VCO}}_{2} }}{\text{slope}}}} - 4.016 + 0.255 \times \ln_{{{\text{age}}\left( {{\text{years}}} \right)}} }}{0.123}$	469	0.19	− 1.67	− 0.03	1.62
OUES	Girls	${\text{Z - score}} = \frac{{\ln_{{{\text{OUES}}}} - 0.4262 - 1.1554 \times \ln_{{{\text{height}}\left( {{\text{cm}}} \right)}} - 0.3173 \times \ln_{{{\text{weight}}\left( {{\text{kg}}} \right)}} }}{0.1906}$	301	0.57	− 1.67	− 0.03	1.61
	Boys	${\text{Z - score}} = \frac{{\ln_{{{\text{OUES}}}} + 3.1486 - 2.0259 \times \ln_{{{\text{height}}\left( {{\text{cm}}} \right)}} - 0.1520 \times \ln_{{{\text{weight}}\left( {{\text{kg}}} \right)}} }}{0.1848}$	355	0.67	− 1.71	− 0.02	1.62
VT_max_ (L)	Girls	${\text{Z - score}} = \frac{{\ln_{{{\text{VTmax}}\left( {\text{L}} \right)}} + 12.1409 - 2.3285 \times \ln_{{{\text{height}}\left( {{\text{cm}}} \right)}} - 0.17293 \times \ln_{{{\text{weight}}\left( {{\text{kg}}} \right)}} }}{0.17085}$	432	0.72	− 1.51	− 0.04	1.64
	Boys	${\text{Z - score}} = \frac{{\ln_{{{\text{VTmax}}\left( {\text{L}} \right)}} + 13.4373 - 2.6582 \times \ln_{{{\text{height}}\left( {{\text{cm}}} \right)}} - 0.0998 \times \ln_{{{\text{weight}}\left( {{\text{Kg}}} \right)}} }}{0.1727}$	477	0.78	− 1.68	0.00	1.61
RR_max_ (1/min)	Girls	${\text{Z - score}} = \frac{{{\text{RR}}\left( {1/\min } \right) - 73.62 + 0.161 \times {\text{height}}\left( {{\text{cm}}} \right)}}{9.56}$	428	0.05	− 1.54	− 0.05	1.58
	Boys	${\text{Z - score}} = \frac{{\ln_{{RR\left( {1/\min } \right)}} - 4.42 + 0.133 \times \ln_{{{\text{weight}}\left( {{\text{kg}}} \right)}} }}{0.194}$	467	0.07	− 1.72	0.04	1.47
BR (%)	Girls	${\text{Z - score}} = \frac{{{\text{BR}}\left( \% \right) - 28.70}}{15.48}$	432	–	− 1.85	0.07	1.56
	Boys	${\text{Z - score}} = \frac{{{\text{BR}}\left( \% \right) - 24.31}}{13.71}$	476	–	− 1.77	0.05	1.51
VEqCO_2max_	All gender	${\text{Z - score}} = \frac{{\ln_{{{\text{VeqCO}}_{2} }} - 3.421}}{0.1195}$	909	–	− 1.66	0	1.66
VEqO_2max_	Girls	${\text{Z - score}} = \frac{{\ln_{{{\text{VeqO}}_{2} }} - 3.575}}{0.1602}$	432	–	− 1.56	0.01	1.60
	Boys	${\text{Z - score}} = \frac{{\ln_{{{\text{VeqO}}_{2} }} - 3.544}}{0.1488}$	477	–	− 1.62	− 0.06	1.80

*BR* breathing reserve; *HR*_*max*_ maximum heart rate; *O*_*2*_* pulse*_*max*_ maximum oxygen pulse; *OUES* oxygen uptake efficiency slope; *RER*_*max*_ maximum respiratory exchange ratio; *RR*_*max*_ maximum respiratory rate; *SD* standard deviation; *VAT* ventilatory anaerobic threshold; *VEqCO*_*2max*_ maximum ventilatory equivalent for CO_2_; *VEqO*_*2max*_ maximum ventilatory equivalent for O_2_; *VT*_*max*_ maximum tidal volume

#### Z-Scores of Maximal CPET Parameters

##### Maximum Heart Rate (HR_max_)

A single linear equation valid for both sexes was generated to define HR_max_ Z-scores:

$${\text{HR}}_{\max } Z{\text{ - score}} = \frac{{\left( {{\text{HR}}_{\max } \left( {{\text{bpm}}} \right) - 188.1} \right)}}{8.9}$$

In the overall cohort, a HR_max_ value of 173 bpm corresponded to the 5th percentile.

##### Maximum Respiratory Exchange Ratio (RER_max_)

For boys, the mathematical model using natural logarithms of RER_max_, height, and weight was the best fit for the data. This equation determined 16% of the variability of the RER_max_ logarithm (*R*^2^ = 0.16). For girls, the mathematical model using RER_max_ without transformation and age was the best fit for the data. This equation determined 14% of the variability of the RER_max_ (*R*^2^ = 0.14). The correlation between predicted and observed RER_max_ using the Z-score model is illustrated in Fig. 1 (panel A). In children with a normal weight, RER_max_ values of 1 in girls and 1.01 in boys corresponded to the 5th percentile (Additional file 1: Table S3).

##### Maximum Workload (Workload_max_)

For both sexes, the mathematical model using natural logarithms of workload_max_, height, and age was the best fit for the data. These equations determined 60% of the variability of the workload_max_ logarithm (*R*^2^ = 0.60) in girls and 76% (*R*^2^ = 0.76) in boys. The correlation between predicted and observed workload_max_ using the Z-score model is illustrated in Fig. 1 (panel B).

##### Maximum Oxygen Pulse (O_2_ Pulse_max_)

For both sexes, the mathematical model using natural logarithms of O_2_ pulse_max_, weight, and height was the best fit for the data. These equations determined 69% of the variability of the O_2_ pulse_max_ logarithm (*R*^2^ = 0.69) in girls and 77% (*R*^2^ = 0.77) in boys. The correlation between predicted and observed O_2_ pulse_max_ using the Z-score model is illustrated in Fig. 1 (panel C).

#### Z-Scores of Submaximal CPET Parameters

##### Ventilatory Anaerobic Threshold (VAT)

For both sexes, the mathematical model using natural logarithms of VAT, height, and weight was the best fit for the data. These equations determined 54% of the variability of the VAT logarithm (*R*^2^ = 0.54) in girls and 68% (*R*^2^ = 0.68) in boys. The correlation between predicted and observed VAT when using the Z-score model is illustrated in Fig. 2 (panel A). In children with a normal weight, a %-predicted VAT value of 55% corresponded to the 5th percentile Additional file 1: Table S3).

##### VE/VCO_2_ Slope

For both sexes, the mathematical model using natural logarithms of VE/VCO_2_ slope and age was the best fit for the data. These equations determined 6% of the variability of the VE/VCO_2_ slope logarithm (*R*^2^ = 0.06) for the girl and 19% (*R*^2^ = 0.19) for the boys. The correlation between predicted and observed VE/VCO_2_ slopes using the Z-score model is illustrated in Fig. 2 (panel B). In children with a normal weight, a VE/VCO_2_ slope value of 37.6 corresponded to the 95th percentile, overall (Additional file 1: Table S3), with a decrease with age from a VE/VCO_2_ slope value of 40.7 in young children aged 5 to 8 years, to a VE/VCO_2_ slope value of 35.9 in adolescents aged 15 to 18 years.

##### OUES

For both sexes, the mathematical model using natural logarithms of OUES, height, and weight was the best fit for the data. These equations determined 57% of the variability of the OUES logarithm (*R*^2^ = 0.57) for girls and 67% (*R*^2^ = 0.67) for boys. The correlation between predicted and observed OUES using the Z-score model is illustrated in Fig. 2 (panel C). In children with a normal weight, OUES_kg_ values of 28.9 in girls and 38.9 in boys corresponded to the 5th percentile (Additional file 1: Table S3).

#### Z-Scores of Ventilatory CPET Parameters

##### Maximum Tidal Volume (VT_max_)

For both sexes, the mathematical model using natural logarithms of VT_max_, height, and weight was the best fit for the data. These equations determined 72% of the variability of the VT_max_ logarithm (*R*^2^ = 0.72) for girls and 78% (*R*^2^ = 0.78) for boys. The correlation between predicted and observed VT_max_ using the Z-score model is illustrated in Fig. 3 (panel A).

##### Maximum Respiratory Rate (RR_max_)

For girls, the mathematical model using RR_max_ and height was the best fit for the data. This equation determined 5% of the variability of the RR_max_ logarithm (*R*^2^ = 0.05). For boys, the mathematical model using natural logarithms of RR_max_ and weight was the best fit for the data. This equation determined 7% of the variability (*R*^2^ = 0.07). The correlation between predicted and observed RR_max_ using the Z-score model is illustrated in Fig. 3 (panel B). In children with a normal weight, RR_max_ values of 67/min for girls and 69/min for boys corresponded to the 95th percentile.

##### Breathing Reserve

This parameter was not influenced by anthropometric variables. The median value of breathing reserve was 30% [0%; 52%] for girls and 25% [0%; 45%] for boys.

##### Maximum Ventilatory Equivalent for CO2 (VEqCO_2max_)

No significant sex differences were found for VEqCO_2max_ and this parameter was not influenced by anthropometric variables. The median value of VEqCO_2max_ was 30.6 [25.1; 37.3].

##### Maximum Ventilatory Equivalent for O_2_ (VEqO_2max_)

This parameter was not influenced by anthropometric variables, except for sex. The median value of VEqO_2max_ was 35.8 [27.9; 46.0] for girls and 34.3 [27.2; 45.0] for boys.

## Discussion

From a cohort of 909 healthy children aged 5–18 years who underwent a cycle ergometer CPET, this study defined paediatric reference Z-score values of the main paediatric CPET parameters, apart from VO_2max_, whose reference values were recently reported by our group [11]. These paediatric CPET reference values were generated using the best mathematical model (linear, quadratic, or polynomial) for the Z-score construction, according to the main anthropometric predictors (sex, age, height, weight, and BMI), to apply to normal and extreme weights. This paediatric cohort is representative of the general contemporary paediatric population, with a balanced sex ratio and a 25%-proportion of overweight or obese children [31], and had recently undergone external validation from a CPET cohort of 232 German and American healthy children [11]. This study intended to satisfy a high methodological quality level and fulfilled 11 of the 14 criteria from the ATS/ACCP statement on cardiopulmonary exercise testing [19]. Unsurprisingly, sex differences were observed in most CPET parameters (apart from maximum heart rate and VEqCO_2max_), resulting in Z-score models generated for boys and girls separately.

In terms of maximal CPET parameters, the existence of Z-scores for the main maximum parameters will improve discussions on maximality criteria in paediatric CPET. The historic equations on predicted maximum heart rate ([220–age]; or 0.65 × [210–age]) were adapted from adult CPET studies and the commonly used value of 80% of predicted maximum heart rate to define maximum exercise was set arbitrarily [19, 32]. Our data confirmed that maximum heart rate was not significantly influenced by age in the paediatric population. Similarly, different cut-off values of maximum RER have been used to define maximal exercise in paediatrics (> 1.05 or > 1.1) [19, 33], but the youngest healthy children often do not reach an RER of 1.05. In our cohort, 10% of subjects had a maximum RER between 1 and 1.05, and 15% between 1.06 and 1.1, most of which (78%) were aged < 12 years. A similar value of RER around 1 at maximal exercise in children has been previously reported [23]. These data confirm that it is probably not appropriate to use RER in maximality criteria for paediatric exercise testing. This study also provides reference values for maximum oxygen pulse, a determinant of aerobic capacity which can be impaired in children with cardiac disease, as in Fontan circulation with limited preload [34]. The maximum oxygen pulse equation will help the clinician to better define the lower normal limit for this important cardiac parameter.

Moreover, the existence of paediatric reference values for submaximal CPET parameters will be useful to interpret cardiopulmonary fitness in children with serious chronic diseases. We found that the reference values of VAT were determined by natural logarithms of height and weight. The VAT is an indicator of aerobic fitness, useful for exercise prescription, especially in cardiac rehabilitation programs to monitor the effect of physical training [7, 35, 36]. In adult studies, wide range of normal values for VAT from 35 to 80% of the predicted VO_2max_ have been reported [19]. Furthermore, percent-predicted VAT values from 50 to 60% observed in adult sedentary subjects are commonly used to define physical deconditioning and patient eligibility for rehabilitation programs. Interestingly, in our study, the 5th percentile of the VAT expressed as a percentage of the predicted VO2_max_ was at 55%, which is exactly the cut-off value used in the QUALIREHAB randomized controlled trial to define children with CHD eligible for cardiac rehabilitation [7]. Furthermore, in this study, the reference values of VE/VCO_2_ slope were determined by a natural logarithm of age, with normal mean values of 33 in youngest children and 28 in adolescents. These results are consistent with the VE/VCO_2_ cut-off value < 28 previously reported to define normal ventilatory efficiency in paediatric CPET [33]. The VE/VCO_2_ slope increases in pulmonary blood flow maldistribution and its prognostic value has been demonstrated in adult heart failure [37]. The existence of paediatric reference Z-scores for VE/VCO_2_ slope opens up new research perspectives to define the prognostic value of this submaximal parameter in various paediatric chronic diseases involving ventilation/perfusion mismatch. As for the OUES, it was strongly influenced by the natural logarithms of height and weight. This submaximal parameter is classically strongly correlated with VO_2max_ and may be useful in severe conditions compromising the achievement of a maximal CPET and making it difficult to accurately interpret peak VO_2_ [14]. The availability of paediatric reference Z-score values for OUES will therefore facilitate the dissemination of this CPET parameter in routine clinical practice. For example, if we focus on children with normal weight, a submaximal CPET with OUES_Kg_ values < 28.9 in girls or < 38.9 in boys, e.g. corresponding to the 5th percentile, may be suggestive of impaired aerobic fitness.

Finally, the existence of paediatric reference Z-score values for ventilatory exercise parameters will improve the interpretation of paediatric CPET, as exercise ventilation may be affected in many paediatric chronic diseases [3, 4, 38, 39]. Ventilatory exercise parameters interact with each other and should not be interpreted independently [28]. For example, in paediatric asthma, e.g. the most common paediatric chronic disease worldwide, physical aerobic fitness is impaired in a quarter of children, with patterns of physical deconditioning in a third of them [4]. Previous studies have suggested that the hyperventilation syndrome during exercise, well described in adult patients [40], was also present in paediatrics and reflected dysfunctional breathing and poor asthma control [41]. Moreover, our group recently showed that a lower breathing reserve was a predictor of limited physical capacity in children with asthma [4]. Moreover, children with expiratory flow limitation, as in cystic fibrosis, present an abnormal breathing pattern during exercise with rapid shallow breathing, evidenced by a higher RR_max_ and a lower VT_max_ [39]. Nevertheless, overall, exercise ventilatory parameters have been scarcely analysed in children [42, 43]. In addition to the existing Z-scores for paediatric spirometry [44], the Z-scores for ventilatory CPET parameters represent a major advance in the analysis of exercise physiology in children.

## Study Limitation

Reference values were not analysed in function of other parameters influencing physical capacity, such as lean mass, pubertal status, or the level of physical activity, as in the study on paediatric VO_2max_ reference Z-scores [11]. The impact of ethnic variation on body fat and muscle mass has not been investigated because of restrictive regulations on the assessment of ethnicity in paediatric clinical research. Moreover, the children included in this study were not community-based, as we were concerned that if we had drawn lots of schools to identify volunteers to perform a CPET, a large proportion of child volunteers would have been athletes. As previously stated by Cumming, “clinic patients without heart defects probably serve as a better normal control group than children obtained from the school system” [45].

## Conclusions

This study defined paediatric Z-score reference values for the main cycle ergometer CPET parameters, applicable to children of normal and extreme weights. This study intended to satisfy a high methodological quality level, by enrolling a large number of subjects, covering wide age and weight ranges, and fulfilling most criteria on high-quality CPET assessment. In addition to the existing reference values for VO_2max_, providing Z-scores reference values of maximal, submaximal, and ventilatory CPET parameters in the paediatric population should be useful in the follow-up of children with various chronic diseases. Thus, new paediatric research fields are opening up, such as prognostic studies and clinical trials using cardiopulmonary fitness parameters as primary or secondary outcomes.

## Supplementary Information

**Additional file 1**. **Supplementary Table 1.** Enumerates the criteria from the ATS/ACCP statement on high-quality CPET assessment. **Supplementary Table 2.** Reports the best R^2^ values according to CPET and anthropometric parameters. **Supplementary Table 3.** Describes the distribution of CPET parameter values in children of normal weight.

## Data Availability

The data underlying this article will be shared on reasonable request to the corresponding author.

## Abbreviations

ATS: American Thoracic Society
ACCP: American College of Chest Physicians
BMI: Body mass index
BR: Breathing reserve
CPET: Cardiopulmonary exercise test
VCO_2_: Carbon dioxide production
FEV1: Forced expiratory volume in 1 s
FVC: Forced vital capacity
FEV1/FVC: Tiffeneau index
HR_max_: Maximum heart rate
O_2_ pulse_max_: Maximum oxygen pulse
OUES: Oxygen uptake efficiency slope
OUES_kg_: Weight-normalized value of oxygen uptake efficiency slope
RER_max_: Maximum respiratory exchange ratio
RR_max_: Maximum respiratory rate
SD: Standard deviation
VAT: Ventilatory anaerobic threshold
%-Predicted VAT: VAT expressed as a percentage of the predicted VO_2max_
VE: Minute ventilation
VEqCO_2max_: Maximum ventilatory equivalent for CO_2_
VEqO_2max_: Maximum ventilatory equivalent for O_2_
VO_2max_: Maximum oxygen uptake
VT_max_: Maximum tidal volume
