# Supplementary material for: Reference Values of Cardiopulmonary Exercise Test Parameters in the Contemporary Paediatric Population
Source: Sports Med Open. 2023 Aug 1;9:68. doi: 10.1186/s40798-023-00622-3 (PMC10393918; doi:10.1186/s40798-023-00622-3)
Supplement: Supplementary file 1 — Additional file 1. Supplementary Table 1. Enumerates the criteria from the ATS/ACCP statement on high-quality CPET assessment. Supplementary Table 2. Reports the best R2 values according to CPET and anthropometric parameters. Supplementary Table 3. Describes the distribution of CPET parameter values in children of normal weight. [file 40798_2023_622_MOESM1_ESM.pdf]

Reference values of cardiopulmonary exercise test parameters in the contemporary paediatric population

Short title: Paediatric CPET parameters Z-scores

Pascal Amedro, MD, PhD[a,b]*; Stefan Matecki, MD, PhD[c,d]*; Taissa Pereira dos Santos[e]; Sophie Guillaumont, MD[f,g]; Jonathan Rhodes, MD, PhD[h]; Suellen Moli Yin[h], MD; Alfred Hager, MD, PhD[i]; Julia Hock PhD[i]; Gregoire De La Villeon, MD[f,g]; Johan Moreau, MD[c,f]; Anne Requirand[d]; Luc Souilla[c], Marie Vincenti, MD[c,f]; Marie-Christine Picot, MD, PhD[j]; Helena Huguet, MSc[j]; Thibault Mura, MD, PhD[e,k]**; Arthur Gavotto, MD, PhD[c,l]**

[a]Department of Paediatric and Congenital Cardiology, M3C National Reference Centre, Bordeaux University Hospital, 1 Avenue Magellan, 33604, Pessac, France

[b]IHU Liryc, Electrophysiology and Heart Modelling Institute, INSERM 1045, Bordeaux University Foundation, Avenue du Haut Lévêque, 33600, Pessac, France

[c]PhyMedExp, CNRS, INSERM, University of Montpellier, 371 Avenue du Doyen Giraud, 34295, Montpellier, France

[d]Paediatric Functional Exploration Laboratory, Physiology Department, Montpellier University Hospital, 371 Avenue du Doyen Giraud, 34295, Montpellier, France

[e]Department of Biostatistics, Clinical Epidemiology, Public Health, and Innovation in Methodology, Nimes University Hospital, University of Montpellier, Place du Professeur Debré, 30029, Nimes, France

[f]Department of Paediatric and Congenital Cardiology, M3C Regional Reference CHD Centre, Montpellier University Hospital, 371 Avenue du Doyen Giraud, 34295, Montpellier, France

[g]Paediatric Cardiology and Rehabilitation Unit, St-Pierre Institute, 371 Avenue de l'Évêché de Maguelone, 34250, Palavas-Les-Flots, France

[h]Department of Cardiology, Boston Children's Hospital, 300 Longwood Avenue, Boston, MA 02115, Boston, USA

[i]Clinic for Paediatric Cardiology and Congenital Heart Diseases, German Heart Centre, Lazarettstrasse 36, 80636, Munich, Germany

[j]Clinical Research and Epidemiology Unit, Montpellier University Hospital, INSERM-CIC 1411, Clinical Investigation Centre, University of Montpellier, Montpellier, France

[k]INSERM, U1061, Neuropsychiatry: Epidemiological and Clinical Research, University of Montpellier, 39 Av. Charles Flahault, 34090, Montpellier, France

[l]Paediatric Intensive Care Unit, Arnaud de Villeneuve Hospital, Montpellier University Hospital, 371 Avenue du Doyen Giraud, 34295, Montpellier, France

*Both authors contributed equally to this work.

**Both authors contributed equally to this work.

Supplementary Table 1. Criteria from the ATS/ACCP statement on high-quality CPET assessment

Criteria	Description	Current study
Population characteristics		
(1) Subjects are community based	The subjects studied preferably be community bases rather than hospital based.	No
(2) Physical activity	Level of physical activity is reported.	No
(3) Racial groups	No exclusion of different racial groups.	Yes
(4) Smokers	Exclusion of smokers in the sample studied.	Yes
(5) Definition of confidence limits	No lack of definition of de confidence limits for individual or specified characteristics. (Include age, sex, and anthropomorphic considerations).	Yes
Sample size		
(6) Number of subjects tested	The number of subjects tested is sufficiently equal or larger than the appropriately powered sample size, with a uniform distribution of subjects for sex and groups.	Yes
Randomization		
(7) Randomization was applied	The study design includes a randomization process to avoid the potential bias seen when more physically active subjects volunteer for the study.	No
Design:		

(8) Prospective study	A prospective study design	Yes
Quality assurance of equipment and methodologies		
(9) Quality control was applied	Quality was achieved using recommendations contained in the ATS/ACCP guidelines and the CPET protocols in accordance with recommendations specified in the ATS/ACCP guidelines	Yes
(10) Exercise testing protocol and procedures	Exercise testing protocol and procedures are described.	Yes
(11) Results are obtained by either breath-by-breath analysis	Results are obtained by either breath-by-breath analysis or mixing chamber treated in accordance with recommendation contained in the ATS/ACCP guidelines.	Yes
Treatment of data		
(12) CPET result in interval averaged and the peak value reported	CPET result in interval averaged, preferably every 30–60 s (to avoid the noise of shorter interval), and the peak value reported represents the mean of the last-completed stage or of all the data collected during the final stage, but preferably for no less than 30 s.	Yes
Validation		
(13) Reference equations are validated	Reference equations are validated in population other than those used to generate the existing data.	Yes
Statistical treatment of data:		

(14) The function that most accurately describes the distribution of the data are used.	The function that most accurately describes the distribution of the data are used. For example, curvilinear (power) functions may more accurately describe the distribution of the data. Furthermore, the precision of the individual and population predicted values are reported	Yes

Legend: ATS, American Thoracic Society, ACCP, American College of Chest Physicians.

Supplementary Table 2. Best R^2 according to CPET and anthropometric parameters.

CPET parameters		Sex	Type of model*	Best R^2			
				Age	Height	Weight	BMI
Maximal parameters	HR_{max} *(bpm)*	All gender	Without transformation	<0.05			
	RER_{max}	Girls	Without transformation	**0.14**[a]	0.12[c]	0.07[c]	0.00[c]
		Boys	Log-Log	0.13[a]	**0.14**[c]	0.07[c]	0.01[c]
	$Workload_{max}$ *(Watt)*	Girls	Log-Log	0.51[a]	**0.58**[a]	0.49[c]	0.20[c]
		Boys	Log-Log	0.65[a]	**0.75**[a]	0.55[c]	0.22[c]
	O_2 $pulse_{max}$ (*mL*)	Girls	Log-Log	0.49[a]	0.62[a]	**0.64**[c]	0.33[c]
		Boys	Log-Log	0.64[c]	**0.76**[a]	0.66[c]	0.33[c]
Submaximal parameters	VAT *(mL/min)*	Girls	Log-Log	0.36[a]	0.44[a]	**0.53**[c]	0.32[c]
		Boys	Log-Log	0.54[c]	**0.66**[a]	0.63[c]	0.33[c]
	VE/VCO_2 slope	Girls	Log-Log	**0.06**[a]	0.06[a]	0.06[c]	0.02[c]
		Boys	Log-Log	**0.19** [a]	0.17 [c]	0.12 [c]	0.4 [c]
	OUES	Girls	Log-Log	0.40[a]	0.50[a]	**0.55**[c]	0.31[c]
		Boys	Log-Log	0.57[b]	**0.67**[a]	0.61[c]	0.28[c]

Ventilatory parameters	VT_{max} *(L)*	Girls	Log-Log	0.61[a]	**0.71[a]**	0.58[c]	0.22[c]
		Boys	Log-Log	0.66[c]	**0.78[a]**	0.63[c]	0.28[c]
	RR_{max} *(/min)*	Girls	Without transformation	0.04[a]	**0.05[a]**	0.04[a]	0.02[a]
		Boys	Log-Log	0.06[a]	0.05[a]	**0.07[a]**	0.05[a]
	Breathing reserve (*%*)	Girls	Without transformation		<0.05		
		Boys	Without transformation		<0.05		
	$VEqCO_{2max}$	All gender	Log-Log		<0.05		
	$VEqO_{2max}$	Girls	Log-Log		<0.05		
		Boys	Log-Log		<0.05		

Legends: CPET, cardio-pulmonary exercise test; HR_{max}, maximum heart rate; O_2 $pulse_{max}$, maximum oxygen pulse; OUES, oxygen uptake efficiency slope; RER_{max}, maximum respiratory exchange ratio; RR_{max}, maximum respiratory rate; VAT, ventilatory anaerobic threshold; $VEqCO_{2max}$, maximum ventilatory equivalent for CO_2; $VEqO_{2max}$, maximum ventilatory equivalent for O_2; VT_{max}, maximum tidal volume. For each anthropometric predictor and each CPET parameter, only the R^2 of the best statistical model is presented among the following models: [a] linear model; [b] quadratic model; and [c] polynomial model. For each CPET parameter, the best anthropometric predictor's R^2 is marked in bold.

Supplementary Table 3. Distribution of CPET parameter values in children of normal weight.

CPET parameters		Sex	N	5th percentile	Median	95th percentile
Maximal parameters	VO_{2max} *(mL/Kg/min)*	Girls	299	29.3	37.9	49.8
		Boys	340	36.1	46.5	56.7
	HR_{max} *(bpm)*	All	639	173	189	203
	RER_{max}	Girls	299	1.01	1.17	1.35
		Boys	340	1.00	1.15	1.31
	$Workload_{max}$ *(Watts)*	Girls	270	75	125	200
		Boys	300	80	140	250
	O_2 $pulse_{max}$ (*mL*)	Girls	299	5.4	8.4	11.9
		Boys	340	6.0	9.5	16.8
Submaximal parameters	VAT *(mL/Kg/min)*	Girls	299	19.0	26.3	37.8
		Boys	340	24.2	32.5	42.3
	%-predicted VAT *(%)*	All	639	55.3	70.5	83.7
	VE/VCO_2 slope	All	639	24	30	37.6

	$OUES_{kg}$	Girls	197	28.9	40.3	55.0
		Boys	242	38.9	49.5	63.8
Ventilatory parameters	VT_{max} *(mL/Kg)*	Girls	299	22.9	29.5	38.9
		Boys	340	24.3	33.4	43.0
	RR_{max} *(/min)*	Girls	296	34.4	49.1	67.3
		Boys	333	36.0	51.0	69.4
	Breathing reserve *(%)*	Girls	299	0.3	29.0	53.0
		Boys	339	0.0	24.0	47.0
	$VEqCO_{2max}$	All	639	25.2	31.0	37.8
	$VEqO_{2max}$	Girls	299	28.4	36.9	47.0
		Boys	340	27.2	34.7	45.3

Legends: HR_{max}, maximum heart rate; O_2 $pulse_{max}$, maximum oxygen pulse; OUES, oxygen uptake efficiency slope; $OUES_{kg}$, weight-normalized value of OUES; RER_{max}, maximum respiratory exchange ratio; RR_{max}, maximum respiratory rate; VAT, ventilatory anaerobic threshold; %-predicted VAT, VAT expressed as a percentage of the predicted VO_{2max}; $VEqCO_{2max}$, maximum ventilatory equivalent for CO_2; $VEqO_{2max}$, maximum ventilatory equivalent for O_2; VT_{max}, maximum tidal volume.
